# Deep Brain Stimulation Reveals Emotional Impact Processing in Ventromedial Prefrontal Cortex

**DOI:** 10.1371/journal.pone.0008120

**Published:** 2009-12-07

**Authors:** Albert Gjedde, Jacob Geday

**Affiliations:** 1 Pathophysiology and Experimental Tomography Center, Aarhus University Hospitals, Aarhus, Denmark; 2 Center of Functionally Integrative Neuroscience, Aarhus University, Aarhus, Denmark; Chiba University Center for Forensic Mental Health, Japan

## Abstract

We tested the hypothesis that modulation of monoaminergic tone with deep-brain stimulation (DBS) of subthalamic nucleus would reveal a site of reactivity in the ventromedial prefrontal cortex that we previously identified by modulating serotonergic and noradrenergic mechanisms by blocking serotonin-noradrenaline reuptake sites. We tested the hypothesis in patients with Parkinson's disease in whom we had measured the changes of blood flow everywhere in the brain associated with the deep brain stimulation of the subthalamic nucleus. We determined the emotional reactivity of the patients as the average impact of emotive images rated by the patients off the DBS. We then searched for sites in the brain that had significant correlation of the changes of blood flow with the emotional impact rated by the patients. The results indicate a significant link between the emotional impact when patients are not stimulated and the change of blood flow associated with the DBS. In subjects with a low emotional impact, activity measured as blood flow rose when the electrode was turned on, while in subjects of high impact, the activity at this site in the ventromedial prefrontal cortex declined when the electrode was turned on. We conclude that changes of neurotransmission in the ventromedial prefrontal cortex had an effect on the tissue that depends on changes of monoamine concentration interacting with specific combinations of inhibitory and excitatory monoamine receptors.

## Introduction

We have shown that activity in a circumscribed region of the medial prefrontal cortex undergoes a change of activity when the region is challenged by administration of the serotonin-noradrenaline reuptake inhibitor clomipramine. In this previous study (File S1), the change of activity in each subject correlated inversely with the emotional impact of standardized emotive images presented to the subjects in separate sessions [1]. In that study, we proposed that the inverse relation is linked to differential distributions of inhibitory and excitatory monoamine receptors in this region. Because of the close relations between the serotonergic, noradrenergic, and dopaminergic systems, we further speculated that a general mechanism would link monoaminergic tone and emotional reactivity in this specific region.

Serotonergic, noradrenergic and dopaminergic mechanisms interact closely in the frontal cortex. Stimulation of the majority of serotonergic receptors (5-HT_1A_, 5-HT_1B_, 5-HT_2A_, 5-HT_3_ and 5-HT_4_) receptors facilitates dopamine (DA) release, with the exception of the 5-HT_2C_ receptors that strongly inhibit DA release in the ventral tegmental area but have no effect on DA release in the frontal cortex [2], [3]. In the frontal cortex, the serotonin receptors facilitate the release of dopamine from the terminals of fibres that arise in the midbrain [4]. We speculate that the effects of this dopamine release depend on the differential distributions of D_1_ and D_2_ receptors that respectively facilitate and inhibit the excitability of the neurons on which these receptors reside.

Previously, we showed that patients with Parkinson's disease display certain forms of disordered cognition when they are treated with deep brain stimulation (DBS) [5]. Here we test the hypothesis that an alleged modulation of monoaminergic tone in patients with Parkinson's disease would reveal the level of emotional reactivity of neurons in ventromedial prefrontal cortex, in a manner similar to that seen during the alleged modulation of serotonergic and noradrenergic tone that occurs after blockade of serotonin-noradrenaline reuptake sites.

## Methods

To test if emotional reactivity relates to changes of neurotransmission in the prefrontal cortex, we obtained PET images of the effect of DBS of the subthalamic nucleus on the cortical activity in seven patients suffering from Parkinson's disease. The patients were approached by one of the authors (JG) of this study and gave written informed consent to the procedures specified in a protocol approved by the official Central Danish Regional Science Ethics Committee.

**Figure 1:**
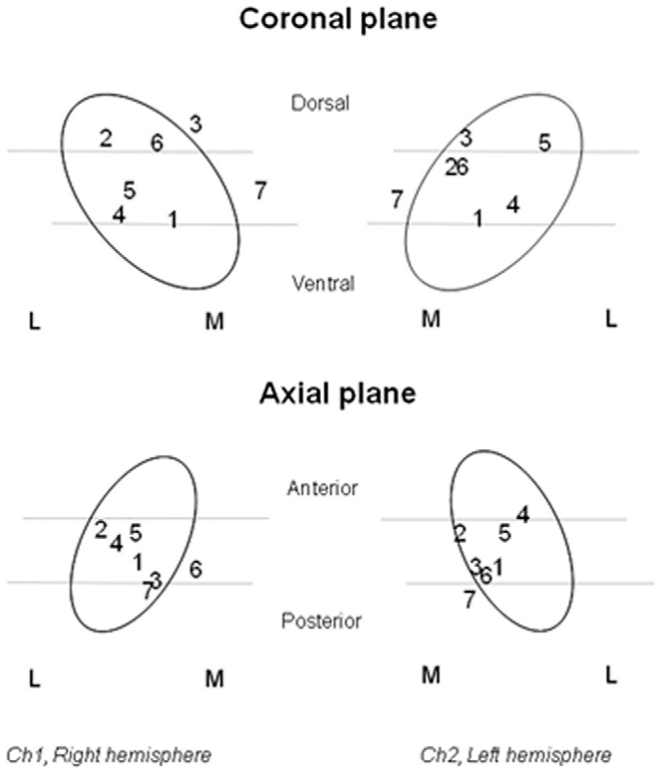
Electrode placement coordinates for the seven subjects studied in this investigation. Electrode positions of the 7 patients entered into the study in the coronal and axial planes.

We correlated the changes of activity in these images with the ratings of emotional impact of standardized emotive images recorded by the patients. Selected details of the patients' condition including medication are given in Table 1. Greater details of the patients' history, treatment and DBS were reported by Geday et al [5]. The positions of the electrodes are shown in Figure 1: The stimulus conditions are given in Table 2, and effects on the UPDRS-III rating scale are given in Table 3.

**Table 1:** Population characteristics and medication.

Subject No.	sex	age (years)	parkinson medication	*Most affected side
1	male	68	pramipexole, levo-dopa	R
2	male	58	pramipexole, levo-dopa	L
3	male	55	pergolide, levo-dopa	L
4	male	65	ropinirole, levo-dopa	R
5	female	66	levo-dopa	R
6	male	53	pramipexole, levo-dopa, entacapone	L
7	male	62	pergolide, levo-dopa	R

* ) Most affected side before STN-operation.

**Table 2:** STN-DBS settings.

Patient	left electrode					right electrode					unipolar
	volt	pulse width (µsec)	rate Hz	µAmp	Ohm	volt	pulse width (µsec)	rate Hz	µAmp	Ohm	
1	3,0	60	195	39	890	3,3	60	200	39	890	yes
2	2,5	60	135	30	670	3,7	60	135	30	1000	yes
3	3,5	60	160	36	945	2,9	60	160	36	780	yes
4	3,5	60	180	36	1042	3,4	60	180	36	1012	yes
5	2,0	60	130	29	532	1,2	60	130	29	670	yes
6	3,3	60	160	30	981	3,3	60	160	30	981	yes
7	2,9	60	170	24	1471	3,2	60	170	42	843	Yes

**Table 3:** STN efficacy measured as difference in motor scores on the Unified Parkinson's Disease Rating Scale (UPDRS III).

Subject No.	UPDRS III “OFF”	UPDRS III “ON”	Effect (ON – OFF)
1	40	22	18
2	18	7	11
3	65	12	53
4	48	8	40
5	14	4	10
6	37	20	17
7	31	12	19
Mean	36,1±17,5	12,1±6,7	24±16,2 (66% improvement)

Details of the PET measurements of cerebral blood flow with oxygen-15-labeled water were given by Geday et al. [5], [6] and Geday and Gjedde [1]. We analyzed the PET images of blood flow on and off the DBS and determined the changes of blood flow elicited by the DBS in a 6 mm radius sphere centred on the Talairach coordinates (1, 55, −13, x,y,z mm), identified as the site of the previously reported interaction between serotonergic-noradrenergic tone and emotional impact [1]. During the PET measurements, the subjects passively viewed the images of the standardized Empathy Picture System (EPS) [7], as described by Geday et al [5].

We correlated the change of blood flow with the emotional impact of the emotive images in the seven patients. We recorded the emotional impact of the images in a separate session with the DBS stimulation off and on, respectively. As in the study reported by Geday and Gjedde [1], we defined the impact as the numerical difference between ratings of unpleasant and pleasant images included in the standardized Empathy Picture System in the separate session with the subjects off and on the DBS.

We analyzed the PET images by voxel-wise regression of PET volumes with local voxel SD estimates against the ratings of emotional impact, as implemented in the Dot statistical parametric mapping of the Montreal Neurological Institute [8], to identify all cortical areas where DBS effects (ΔrCBF/rCBF), defined as the relative rCBF change associated with an intervention or challenge, correlated with emotional impact. We accepted only changes with t>4.5 or t <4.5 (corrected for multiple comparisons) as having reached significance (P<0.05) with 46 degrees of freedom and a search volume 600 cm^3^ for all cortical gray matter at an FWHM of 12 mm.

In addition to the global analysis of all cortical gray matter (excluding the cerebellum), we restricted a search to a volume of interest (VOI) consisting of one 6-mm-radius sphere centered on a site in the right inferior medial prefrontal cortex (IMPC) previously shown to be deactivated by emotional content [1], [5]–[7] at the Talairach coordinates (x, y, z mm) 1, 55, −13. Threshold t-statistics of the restricted search was 3.46 for P<0.05 with 46 degrees of freedom with a search volume of 0.984 cm^3^ at an FWHM of 12 mm.

## Results

**Figure 2:**
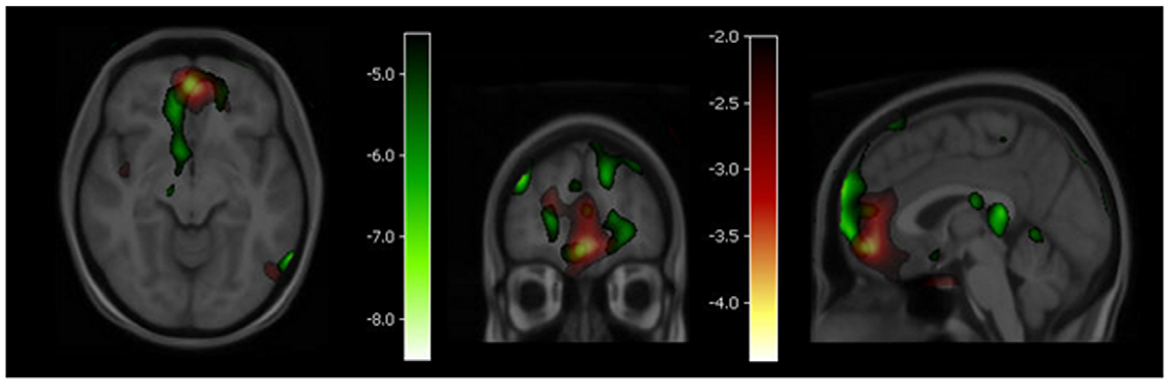
Sites of significant correlation of emotional impact and blood flow changes by deep brain stimulation. Green scale: Areas in the cortex with significant correlation of emotional impact and blood flow change elicited by STN-DBS. A region centred on Talairach coordinates 1, 55, −13, (x,y,z mm) is marked by yellow circle. The threshold in the images is P<0.05 for t<−4.50. Red scale: Areas in the frontal cortex with an interaction between emotional reactivity and blood flow changes elicited by a clomipramine challenge [1]. The area of significant interaction (threshold P<0.05 for t<−3.46) in BA11 partially coincides with the area of interaction between STN-DBS and emotional impact.

We completed the regression analysis of blood flow changes elicited by STN stimulation against emotional impacts ratings, equal to the difference between ratings of pleasant images and ratings of unpleasant images, on a scale from −3 to +3 in the seven subjects. The regression revealed several sites in which the correlation between the two measures reached significance (P<0.05, corrected for multiple comparisons). The correlation is shown as negative in Figure 2, indicated by the negative t-value bars. The negative correlation implies that increases of blood flow occur at the low end of the emotional impact ratings.

**Figure 3:**
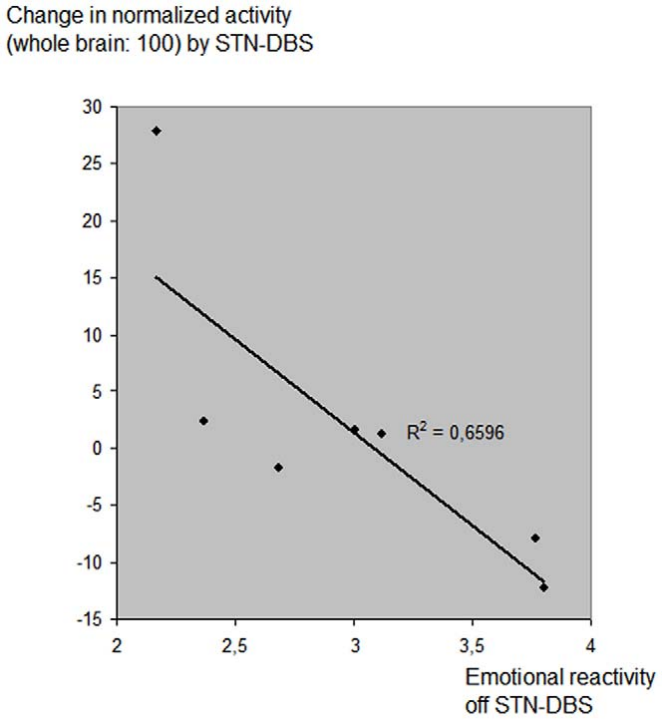
Significant change of blood flow in response to deep brain stimulation. Changes of blood flow at site in ventromedial prefrontal cortex in relation to separately recorded emotional impact. Abscissa: Individual emotional reactivity scores (ratings of pleasant images minus ratings of unpleasant images from the EPS). Ordinate: Blood flow change in 6-mm sphere centred at Talairach coordinates (1, 55, −13, x,y,z mm) elicited by STN-DBS.

One of the sites at which blood flow underwent this change associated with the DBS in correlation with emotional impact coincided with the area at which blood flow equally underwent a change during the clomipramine challenge reported by Geday and Gjedde [1], in both cases in inverse proportion to the emotional impact of the emotive images presented in the absence of a drug challenge or electrical stimulation. This previously reported site is in the region of Brodmann's Area (BA) 11, centred on the Talairach coordinates (1, 55, −13 [x,y,z] mm),

We specifically determined the change of relative blood flow values at this site in a specific volume-of-interest with a radius of 6 mm, centred on the previously identified coordinates, as shown in Figure 1. These changes are listed in Table 4, together with the ratings of emotional impact with the electrodes off and on. When we compared the blood flow changes in this region with the ratings of emotional impact, we found significant correlation (P = 0.027) between the blood flow change elicited by the DBS and the ratings of emotional impact with the DBS off, as shown in Figure 3. The same comparison with the ratings of emotional impact with the DBS on did not quite reach significance (P = 0.12). With the mean of the off and on ratings, the significance increased further (P = 0.019). While this implies that the change of emotional impact ratings with stimulation failed utterly to reach significance (P = 0.94), we note that the ratings of emotional impact on and off DBS in five of the seven subjects changed in parallel with the change of blood flow at this site, relative to whole-brain. These five subjects are marked with an asterisk in Table 1.

**Table 4:** Emotional reactivity and normalized brain activity.

subject	emotional reactivity		change	activity (whole brain average 100)		change
	off STN-DBS	on STN-DBS		off STN-DBS	on STN-DBS	
1	3.80	3.65	−0.15*	102.811	90.700	−12.111*
2	3.77	2.79	−0.98*	132.000	124.214	−7.786*
3	2.69	2.30	−0.39*	118.469	116.799	−1.670*
4	3.12	3.07	−0.05	127.382	128.639	1.258
5	3.00	3.70	0.70*	116.846	118.504	1.658*
6	2.37	3.37	1.00*	108.837	111.234	2.397*
7	2.17	1.89	−0.28	89.130	117.006	27.876

## Discussion

The results support the predicted link between the emotional impact of emotive images and changes of activity in the prefrontal cortex associated with specific drug challenge or electrode stimulation. Thus, in subjects with a low emotional impact, activity measured as blood flow at a site in the ventromedial prefrontal cortex rose in patients with Parkinson's Disease and DBS electrode turned on, as it did in healthy subjects challenged with a serotonin-noradrenaline reuptake inhibitor, while in patients of high impact, the activity at this site in the ventromedial prefrontal cortex declined when the electrode was turned on, as it did in healthy subjects of high impact challenged with the drug. We explain the findings in both groups by the claim that monoamines (serotonin, noradrenaline, or dopamine) are released from terminals in the ventromedial prefrontal cortex and that this release has an effect on the emotional reactivity that depends both on the monoamine concentration and on the relative distributions of inhibitory and excitatory dopamine receptors.

In the previous study [1], we considered the theoretical consequences of the well-known presence of two major populations of monoaminergic receptors, of which one class is inhibitory and the other class is excitatory. Examples include the serotonin receptors among which the 5HT_1A_ receptors mediate lower excitability and most other serotonin receptors mediate higher excitability of the cells where they reside, and the dopaminergic receptors among which the D_1_ receptors when occupied contribute to raised excitability and the D_2_ receptors when occupied lower the excitability of the cells on which they reside. The direction of the effect (inhibitory or excitatory) of an increment of endogenous ligand occupation then depends on the baseline ligand concentration from which the increment occurs. At low concentrations, the effect of an increment is in one direction, depending on the distribution of excitatory and inhibitory receptors. At concentrations above a certain threshold, the effect of the increment is in the opposite direction. The net effect of the transmitter release therefore reflects the pre-existing transmitter concentration in relation to the receptor distribution, as well as the magnitude of the increment.

Geday et al. [5] reported the results of a different setup in which patients off medication and stimulation, who passively viewed emotionally pregnant scenes relative to neutral scenes had significantly less activation of the right inferior temporal cortex and significantly greater activation of the left anterior cingulate gyrus than healthy volunteers. The result refers to an effect of the emotional impact on the measured change of blood flow in PD compared to control. Although there is no blood flow measurement in the present study in conjunction with the rating of the images, the 2006 findings in relation to the present findings could mean that DBS of the STN restores a habitual sensitivity by reactivation of affected mechanisms of emotional impact processing.

What is the evidence that there is monoamine release in the ventromedial prefrontal cortex or elsewhere during DBS? One PET study of the binding of the D_2_ receptor radioligand raclopride in the striatum [9] did not give evidence consistent with increased extracellular dopamine in the striatum during stimulation of the subthalamic nucleus (STN-DBS) in humans. However, other studies strongly indicate that STN-DBS does increase the release of dopamine, measured either directly by means of microdialysis [10], [11] or indirectly by means of raclopride and PET [12]. None of these studies provided direct evidence of dopamine release in the ventromedial prefrontal cortex, where the density of dopamine receptors is too low to accurately record the changes of binding with raclopride.

However, considerable circumstantial evidence links dopamine release in the prefrontal cortex to the regulation of emotive and cognitive functions subserved by this part of the brain. In rats, Bean et al. [13] used microdialysis to measure extracellular concentrations of dopamine and neurotensin in the rat prefrontal cortex during electrical stimulation of the median forebrain bundle. The stimulation released both dopamine and neurotensin. In rat prefrontal cortex, dopamine strongly modulates long-term depression (LTD) and potentiation (LTP) of glutamatergic synapses. Otani et al. [14] reported that the dopaminergic facilitation of LTD in part is triggered by dopamine receptor activation. Lewis et al. [15] confirmed that stimulation of the ventral tegmental area leads to states of depolarization of pyramidal neurons in the prefrontal cortex that are blocked by antagonists of the D_1_ receptors, indicating that the increased excitability is due to release of dopamine. During high frequency stimulation of the STN in rats, intracellular recordings from dopaminergic neurons in substantia nigra pars compacta by Lee et al. [16] revealed increased generation of EPSPs and increased frequency of action potentials. In patients with Parkinson's disease, Jahanshahi et al. [17] observed that DBS of the STN improved cognitive skills assessed by standard neuropsychological tests. In three patients with treatment resistant depression implanted with bilateral DBS electrodes in the nucleus accumbens, significant changes of brain metabolism were observed in frontostriatal networks, associated with improvements of anhedonia which is a key deficiency in refractory major depression [18].

Together these studies provide a strong basis for the claim that DBS of the STN directly or indirectly leads to release of monoamines in the ventromedial prefrontal cortex, the effect of which is contingent upon the quantity of monoamines released and the relative densities of excitatory and inhibitory monoamine receptors, with important consequences for the emotional impact of emotive sensations.

## Supporting Information

File S1 Related paper by authors. This is a recently published paper to which the submitted ms refers
(0.24 MB PDF)
