# Supplementary material for: Deep Brain Stimulation Reveals Emotional Impact Processing in Ventromedial Prefrontal Cortex
Source: PLoS One. 2009 Dec 7;4(12):e8120. doi: 10.1371/journal.pone.0008120 (PMC2781158; doi:10.1371/journal.pone.0008120)
Supplement: File S1 — Related paper by authors. This is a recently published paper to which the submitted ms refers (0.24 MB PDF) [file pone.0008120.s001.pdf]

Monoaminergic Modulation of Emotional Impact in the Inferomedial Prefrontal Cortex

JACOB GEDAY[1,2]* AND ALBERT GJEDDE[1]
[1]PET Center and Center of Functionally Integrative Neuroscience, Aarhus University, Aarhus, Denmark
[2]Department of Neurology, Aarhus University Hospitals, Aarhus, Denmark

KEY WORDS monoamines; inferomedial prefrontal cortex; BA11; emotional reactivity; visual stimuli

ABSTRACT People assess the impact of emotionally loaded images differently. We define this impact as the average difference between individual ratings of standardized "pleasant" and "unpleasant" images. To determine the neuroanatomical correlate of a hypothetical interaction between emotional impact and cerebral excitability, we first determined the individual effect on cerebral blood flow of a pharmacological challenge with the monoamine reuptake inhibitor clomipramine in nine healthy volunteers. In a later, independent, session the nine volunteers rated pleasant, neutral, and unpleasant images of the standard Empathy Picture System on a scale from +3 to −3. We then used regression analysis to identify sites in the ventromedial prefrontal cortex at which the two separately acquired measures, blood flow change and emotional impact of images, correlated significantly. The regression analysis identified a locus in Brodmann's area 11 of the inferomedial prefrontal cortex (IMPC) at which these two separate measures had significant inverse correlation. Thus, under the specific circumstance of positron emission tomography (PET) of a pharmacological challenge, a key region of the inferomedial prefrontal cortex underwent deactivation in proportion to a separately rated emotional impact of a stimulus. We propose a specific pharmacodynamic mechanism that explains the correlation between the emotional impact and the effect of a serotonin-noradrenaline reuptake inhibitor on cerebral blood flow. **Synapse 63:160–166, 2009.** © 2008 Wiley-Liss, Inc.

INTRODUCTION

It is an everyday observation that people tend to perceive emotive events differently. For instance, weddings or funerals are perceived as very emotional by some and as very trivial by others. The impact varies from person to person, and thus we define emotional impact as the subjective valence that people assign to an emotional experience. In previous work, we noted that different volunteers assign different valences to the same emotive images of the standard Empathy Picture System (EPS, Table A, online material, Geday et al., 2003, 2006, 2007). A similar observation was made by Harvey et al. (2007a). Regardless of the valence of the images, some subjects tend to rate the emotive EPS images as more emotive than other subjects do. When the former rate the positive and negative EPS series, they rate the positive images on average as more pleasant and the negative as more unpleasant than the latter do, on a scale from −3 to +3. In the following, we seek a neurobiological explanation for this difference.

Increasing evidence relates the emotional impact of a stimulus to individual differences of the strength of monoaminergic neurotransmission in the prefrontal cortex: In affective disorders, patients display inappropriate reactions to external inputs (Lesch and Mossner, 1998), and compared to healthy subjects, patients recovered from depression have significantly higher 5-HT_{2A} receptor binding potentials in the orbitofrontal cortex (Bhagwagar, 2006), consistent with decreased monoaminergic neurotransmission (Duman, 2004), or upregulation of 5-HT_{2A} receptors, or both. In healthy subjects, variations of the density of specific prefrontal 5-HT receptors appear to be associated with different personality traits. Thus [^{18}F]fluoroethylspiperone bind-

Additional Supporting Information may be found in the online version of this article.

*Correspondence to: Jacob Geday, PET Center and Center of Functionally Integrative Neuroscience, Aarhus University, Noerrebrogade 44, 8000 Aarhus C, Denmark. E-mail: geday@pet.auh.dk

Received 7 March 2008; Revised 8 May 2008; Accepted 3 June 2008
DOI 10.1002/syn.20570
Published online in Wiley InterScience (www.interscience.wiley.com).

© 2008 WILEY-LISS, INC.

ing to the excitatory $5HT_{2A}$ receptors in the frontal cortex is associated with greater tendency to avoid danger (Moresco, 2002). Borg et al. (2003) found that the binding of [^{11}C]WAY100635 to the inhibitory $5HT_{1A}$ receptors correlates inversely with scores of a personality trait known as "self-transcendence." Also, subjects with the α_{2A} adrenergic receptor genotype GG score significantly higher on Depression and significantly lower on Morality and Orderliness scales than subjects with CC and CG genotypes (Mäestu et al., 2008).

Among the serotonin receptors, the subtypes $5HT_{1A}$ and $5HT_{2A}$ dominate in the prefrontal cortex. The $5HT_{1A}$ receptors lower, and the $5HT_{2A}$ receptors raise, glutamatergic excitability. Serotonin has 50–1000 times greater affinity for $5HT_{1A}$ sites than $5HT_{2A}$ sites (Peroutka and Snyder, 1983). Also noradrenaline has at least two receptors of reciprocal action in the prefrontal cortex, the high-affinity inhibitory α_2 adrenoreceptor, and the lower-affinity excitatory α_1 adrenoreceptor, as well as possibly a low-affinity inhibitory β adrenoreceptor (Ramos and Arnsten, 2007).

In the following, we present evidence indicating that the opposite actions of the most common monoamine receptor subtypes establish a regulatory mechanism which controls the net effect of increased monoaminergic neuromodulation by means of the relative densities and affinities of excitatory and inhibitory receptors at the prevailing baseline concentrations of monoamines in the extracellular fluid. This is the claim that the same release of monoamine therefore raises the excitability in some subjects and lowers the excitability in others, depending on the baseline concentration to which the released monoamine is added. The key to this mechanism is the baseline concentration of monoamines when the local densities and affinities are given. A formal kinetic proof of this contention is given in the Appendix.

The densities of prefrontal cortical $5HT_{1A}$ and $5HT_{2A}$ receptors vary considerably among subjects (Adams et al., 2004; Bailer et al., 2005, 2007; Moller et al., 2007), but mostly so for the $5HT_{1A}$ receptors in the IMPC. The region is thought to facilitate attention to emotive stimuli (Geday et al., 2007), but the evidence for this role is equivocal; in some studies the region undergoes activation (Amodio and Frith, 2006; Ochsner and Gross, 2005; Phan et al., 2002) while in other studies it undergoes deactivation (Paradiso et al., 1999; Geday et al., 2003). Indeed, individuals with higher emotional impact of the emotive EPS series during emotional stimulation deactivate the IMPC more than individuals with lower impact (Geday et al., 2003). On the basis of the evidence presented above (varying traits of personality, and varying densities and affinities of reciprocally acting receptors), we claim that the individually varying densities of monoamine receptors with different affinities and reciprocal actions at specific monoamine concentrations explain the equivocal evidence of emotional impact in the IMPC.

To test this claim, we recorded the emotional impact of series of standardized pleasant and unpleasant images from the EPS in nine healthy volunteers. We compared this impact with the ability of a serotonin-noradrenaline (SNRI) reuptake inhibitor in a separate, prior examination of the same subjects (Geday et al., 2005) to change regional cerebral blood flow (rCBF) in a region of the IMPC. This region of the IMPC is the one we previously found to be deactivated by emotional stimulation (Geday et al., 2003). We specifically predicted that individual emotional impact scores would correlate with changes of cerebral activity in the IMPC elicited by an SNRI, measured as blood flow changes.

MATERIALS AND METHODS

Subjects

Nine healthy subjects (two women and seven men, mean age 52 $\pm$ 11 (SD) years) gave written informed consent to participation as approved by the Regional Science Ethics Committee of Central Denmark Region. The age range followed the recommendation of this ethics committee. Subjects were unmedicated and had no known psychiatric or neurological illness. Hamilton depression and anxiety inventories (Bech et al., 1988), self-rating anxiety and depression questionnaire (Bech et al., 1986), and the mini-mental state examination (Folstein et al., 1975) revealed no abnormality of any subject.

Study 1: Pharmacological challenge

Subjects underwent three successive tomographies before, and three after, 30-min i.v. infusion of clomipramine (0.15 mg/kg in 100 ml isotonic saline). Ten minutes interval was given for each successive tomography, except for the infusion intermission that lasted 45 min (30-min infusion followed by 15-min rest). Venous blood samples were obtained for analysis of serum concentrations of clomipramine 60 min after the end of the infusion. The subjects were asked to relax, not to move the head or body, and to keep their eyes open during the 3-min intervals of brain scanning (Raichle et al., 2001). We gave the flow tracer $H_2{}^{15}O$ as a fast bolus in an antecubital vein. The tomography used the ECAT Exact HR47 camera from Siemens/CTI (Knoxville, TN). The forty-seven 3.1-mm sections were filtered to 12 mm FWHM. For anatomical localization of activation sites, T1-weighted magnetic resonance imaging (MRI) was performed. The PET images were coregistered to each individual's MRI. PET and MRI data were mapped into standardized stereotaxic space (Talairach and Tournoux, 1988) using a nine-parameter affine transformation. Subjects were questioned from time to time concerning whether they had sensations that could be attributed to the infusion and a record was kept of their

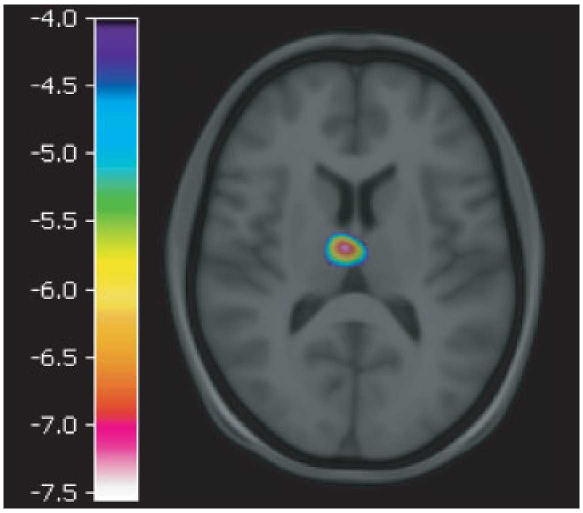

Fig. 1. The infusion condition of clomipramine led to a significant decrease of regional cerebral blood flow in the mediodorsal thalamus, at the site previously reported (Smith and Geday, 2001; Geday et al., 2005). Threshold $P < 0.05$; $t < -5.07$ (No of degrees of freedom: 46, search volume 600,000 mm^3, FWHM 12 mm).

TABLE I. Subject's individual scores of the EPS series

	Emotional value of series			
Subject No.	Neutral	Positive	Negative	Positive-negative
1	0.35	1.01	−0.27	1.28
2	−0.07	0.92	−1.51	2.43
3	0.2	1.01	−1.41	2.42
4	0.6	1.57	−1.4	2.97
5	0.18	1.06	−1.23	2.29
6	−0.13	0.44	−0.73	1.17
7	0.03	1.37	−1.52	2.88
8	0.04	0.76	−0.85	1.61
9	0.7	1.53	−1.14	2.67
avg	0.21	1.07	−1.12	2.19
SD	0.27	0.35	0.40	0.64
Values from the EPS (previous scores of 34 normal, healthy subjects)				
avg	0.32	1.12	−1.62[a]	2.75
SD	0.77	0.83	1.02	

[a]The subjects' scores for the negative images deviated significantly from the normal material $P < 0.01$, only subject 2, 4, and 5 scored within the 5% threshold (+/−0.34).

replies. In addition, obvious changes in their condition were recorded.

Study 2: emotional impact

The standardized routine Empathy Picture System used in this study consists of 12 independent series of images. The validity of valence and the valence medium classifications were established in details in previous studies (Geday et al., 2003, 2006, 2007). In six of the series, the emotional valence primarily is dictated by the facial expressions of the persons depicted, and in the six remaining series, the valence is dictated by the depicted situations. The series of images were further subdivided according to emotional valence (positive, negative, or neutral). The subjects rated each image on a scale from −3 to 3, with −3 as subjectively the most unpleasant, 0 as neutral, and 3 as subjectively the most pleasant. Images were presented as six mixed series of 60 unpleasant, neutral and pleasant images. For all nine subjects, we calculated an emotional impact score, defined as the numerical difference between the averaged individual scores of pleasant and unpleasant image series. The theoretical maximum impact score is therefore 6.

Correlation of studies 1 and 2

We performed pixel-wise regression of PET volumes with the local voxel SD (Worsley et al., 1996), to identify all cortical areas where monoaminergic reactivity (ΔrCBF/rCBF), defined as the relative rCBF change associated with administration of clomipramine, correlated with emotional reactivity. Only sites with changes $t > 3.8$ or $t < -3.8$) were reported. In addition to the global analysis of all cortical gray matter (excluding the cerebellum), we restricted a search to a region of interest (ROI) consisting of two 10-mm-radius spheres centered on a site in the right inferior medial prefrontal cortex (IMPC) previously shown to be deactivated by emotional content (Geday et al., 2003, 2006, 2007) and to its mirror site in the left hemisphere at the Talairach coordinates (x, y, z mm) 15, 51, −8, and −15, 51, −8. Threshold t-statistics of the restricted search was 3.46 for $P < 0.05$ with 46° of freedom, search volume 6.283 cm^3, at an FWHM of 12 mm, whereas threshold t-statistics for the global search with 46 degrees of freedom, search volume 600,000 cm^3, at an FWHM of 12 mm, was 5.07 for $P < 0.05$.

RESULTS

Study 1: Pharmacologic challenge

Thirty minutes after the i.v. infusion, the serum level of clomipramine averaged 74 nM with a standard deviation of 32 nM. Of the nine subjects, two reported nausea 25–35 min after the start of infusion of clomipramine. However, none requested termination of the scanning session, despite reminders that they were of course free to do so. As shown in Figure 1, the parametric map of average blood flow changes in the brains of the nine subjects revealed decreased blood flow in the mediodorsal thalamus at the Talairach coordinates 4, −9, 13 mm compared to flow changes elicited by placebo (reported as Smith and Geday, 2001; Geday et al., 2005). No other site of significant main effect appeared anywhere, including the prefrontal cortex.

Study 2: Emotional impact scores

Among the nine subjects, individual scores of emotional impact varied as shown in Table I. On average they did not differ from the average impact of 2.75 in a separately tested control population of 34 individuals.

TABLE II. Interaction between individual emotional impact of the EPS series and cerebral blood flow changes elicited by SNRI

Anatomical region	BA	Talairach coordinates x	y	z	t-value	P (Corrected for multiple comparisons)
Right medial frontal gyrus	11	1	55	−12	−3.85[a]	<0.02
Left middle temporal gyrus	21	−63	−27	0	−3.99[b]	NS
Left supramarginal gyrus	40	−52	−52	33	−3.83[b]	NS

[a]Search volume in right IMPC 6,283 mm^3 df: 46, FWHM 12 mm, t-threshold $P < 0.05$: 3.46.
[b]Search volume in gray matter 500,000 mm^3 df: 46, FWHM 12 mm, t-threshold $P < 0.05$: 5.01.

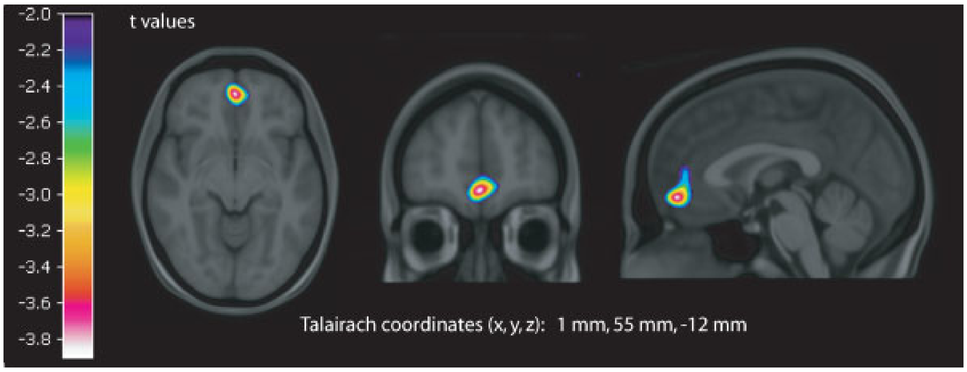

Fig. 2. Neuroanatomical correlate of interaction between the monoaminergic challenge condition and the emotional impact in inferomedical prefrontal cortex. Average T2-weighted MR sagittal section at coordinates $x = 1$ mm, $y = 55$ mm, $z = -12$ mm with PET site of significant activation superimposed, color-scaled as shown by bar on left between $t = -3$ and $t = -4.1$.

Interaction of study 1 and study 2

Unlike the separate analyses of Studies 1 and 2, analysis of interaction between the scores of emotional impact and changes of blood flow in the clomipramine administration condition revealed significant interaction between the individual scores of emotional impact and the change of rCBF in the restricted search at a single site within the ROI in Brodmann's area 11 in the midline of the IMPC (Table II, Fig. 2). In subjects with low scores of emotional impact, the pharmacological challenge condition raised the rCBF, while the rCBF decreased in subjects with high scores of emotional impact (Fig. 3).

The global search revealed insignificant interactions between emotional impact scores and blood flow changes in temporal and parietal areas (Table II) known to be active in emotional processing, and to serve facial recognition and perception of expressions (Iidaka et al., 2001; Phillips et al., 1998), social interaction (Schilbach et al., 2006), and assessment of anger in faces and gestures (Grosbras et al., 2006).

DISCUSSION

In this study, we demonstrate that the effect of a simple pharmacological challenge condition on blood flow covaries significantly with the magnitude of reaction to an emotive visual stimulus This covariation takes place in a particular place in the neocortex, previously demonstrated to be significantly less active during emotional stimulation.

Monoaminergic modulation of particular targets in the inferomedial prefrontal cortex is known to serve the processing of emotions and attention. The covariation of emotional impact and blood flow in the pharmacological challenge condition demonstrated in the present paper occurred in a place of considerable individual variability of especially $5HT_{1A}$, but also of $5HT_{2A}$ receptor subtype densities. With the actual values published by Bailer et al. in 2007 for the density of $5HT_{1A}$ and $5HT_{2A}$ receptors in the part of the IMPC reported here, the coefficient of variation of the $5HT_{1A}$ receptors is as much as 26.7%, and of the $5HT_{2A}$ receptors 17.1%. In the present study we did not directly map serotonin receptors, but the study of Bailer et al. substantiates the individual differences of the density of $5HT_{1A}$ receptors that are likely to be present also in the subjects studied here.

As no completely selective PET adrenoceptor tracers yet exist for human in vivo studies, the neuroimaging evidence of the functions of noradrenaline is sparse compared to that of serotonin. Noradrenergic innervation in general is important to the normal cognitive function of the prefrontal cortex. Noradrenergic α_2 agonists improve the performance of tasks that challenge the prefrontal cortex, but have little effect on tasks that do not, with the opposite being the case for noradrenergic α_1 stimulation (Ramos and Arnsten, 2007). Less is known about the effect of noradrenaline

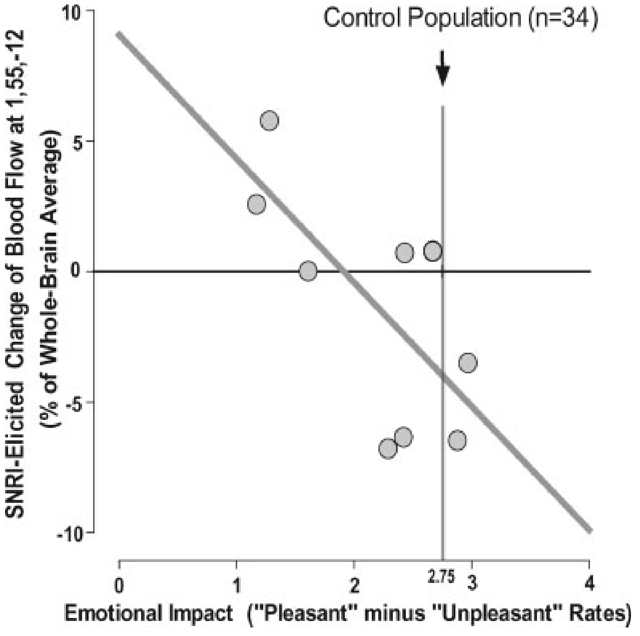

Fig. 3. Regression analysis of monoaminergic regulation of emotional impact at coordinates $x = 1$ mm, $y = 55$ mm, $z = -12$ mm shown as linear regression of percentage changes of rCBF indices (ordinate) vs. individually averaged emotional impacts (abscissa) as defined in text.

on emotional perception, but polymorphism of the gene for the α_{2C} adrenoreceptor appears to have an impact on how images of sad faces are being processed in the subgenual cingulate as well as in the amygdala (Neumeister et al., 2006).

We suggest that the conundrum of multiple receptor subtypes serving the same transmitter in the same region of the brain is explained by the concentration-dependent differential reactivity established in places where receptor subtypes of different density, affinity, and action (inhibitory and excitatory) coexist, as described in details in the appendix.

In addition to differences of receptor densities and affinities, the differential reactivity can also reflect local differences of baseline monoamine concentration. That this is a possibility is supported by evidence of monoamine transporter heterogeneity in mammalian brain. Thus, the frequencies of the "long" (l) and "short" (s) serotonin transporter (SERT) (5-HTT) gene alleles vary in relation to traits of personality (Greenberg et al., 1996), possibly because the 5-HTT mRNA and 5-HT reuptake capacity both are higher in cells with two copies of the l allele (l/L) than in cells with one (l/s) or two copies (s/s) of the s allele (Lesch et al., 1996). Conversely, Graff-Guerrero et al. (2005) demonstrated that glucose metabolism in Brodmann's Areas (BA) 10 and 11 of the prefrontal cortex (IMPC) is higher in people with two copies of s (s/s) than in people with one or two copies of l (l/L and s/L). However, in the present study we did not assess the SERT (5-HTT) genotype of the subjects.

The site of interaction in the IMPC reported here is close to sites where previously reported effects of a serotonergic challenge were held to be associated with disorders of personality. Soloff et al. (2000) found that the glucose metabolic rate in patients with borderline personality disorder responds less than in normal volunteers to fenfluramine, in areas of the prefrontal cortex associated with regulation of impulsive behavior, as well as in the middle temporal gyrus. Also, the site of the present IMPC deactivation is next to the site of deactivation imposed by fenfluramine in BA 32 in depressed subjects, in whom Kegeles et al. (2003) described a correlation between greater metabolic suppression and acute mood improvement during the challenge. In a study of depressed patients with and without borderline personality disorder, fenfluramine affected the metabolism in BA 11, 21, and 40 in a manner that depended on the presence of the personality disorder (Oquendo et al., 2005). Thus, in the IMPC, monoaminergic reactivity correlates with personality disorder and depression.

In healthy subjects the IMPC is involved in the processing of emotional stimuli (Amodio and Frith, 2006; Phan et al., 2002), attention (Geday et al., 2003, 2007; Raichle et al., 2001), and social perception (Geday et al., 2003; Harvey et al., 2007b). In the present study, we focused on emotional reactivity, because the IMPC as ROI is deactivated by attention to an emotional stimulus (Geday et al., 2003, 2007). We suggest that a similar relationship may exist between monoaminergic reactivity and individual differences in other cognitive functions served by the medial prefrontal cortex; from outcome monitoring high in the anterior cingulate to reward evaluation deep in the orbitofrontal cortex (Amodio and Frith, 2006).

Methodological considerations and limitations

The comparison of flow change to emotional impact scores interestingly implies that the average emotional impact recorded in a control population of 2.75 signifies an average decline of rCBF in the population as a whole (Fig. 3). However, the present subjects tested with the standard EPS on average had a slightly lower score of emotional impact than the much larger group of normal volunteers. We regard this difference as a coincidental result of the lower number of subjects in the present study, which specifically addressed the meaning of variable impact scores. As we focus on an interaction rather than a main effect of emotion, the lower impact scores do not represent a potential bias to our conclusion. The conclusion is based on the result of a robust regression (Fig. 3). The uneven distribution of gender and the

variability of age do not confound the results, as neither covaries with SNRI reactivity, nor constitutes a main effect in itself.

The biological half-life of clomipramine is 12–40 h and 54–77 h for its active metabolite; desmethylclomipramide. To perform the PET scanning in one session, all subjects received clomipramine before the last three scans in the PET session, inevitably leading to a potential systematic order bias, as the flow changes could be a consequence of anxiety elicited by the administration of a pharmacological substance, rather than as an effect of the substance itself. If such an effect were present, it would be most pronounced in the first scan after the drug administration when anxiety is greatest, and would be expected to wear off during the two next scans. To investigate this timing effect, we did an additional regression analysis of the three last scans only. This analysis revealed no effect of order at any time.

The majority of previously published studies of visually evoked emotion used the International Affective Picture System (Lang et al., 2005) in which images are evaluated on two axes; valence and arousal, both ranging from 1 to 9, The IAPS rates emotional perception (valence) against emotional experience (arousal), whereas the present standard EPS image system rates only one axis of emotional impact ranging from −3 to +3. However, the distinction between valence and arousal in an experimental setting is theoretical rather than practical. Reported values of valence and arousal from individual images in the IAPS covary as a U-shape with the lowest arousal at valence 5 (neutral, Lang et al., 2005). Thus, ratings of the EPS correspond in principle to the valence/arousal ratings of the IAPS as descriptions of the same emotional response.

ACKNOWLEDGMENTS

The authors thank Donald F. Smith, Center for Psychiatric Research, Aarhus University Psychiatric Hospital, Risskov, Denmark for recruiting and testing the subjects.

REFERENCES

Adams KH, Pinborg LH, Svarer C, Hasselbalch SG, Holm S, Haugbøl S, Madsen K, Frøkjaer V, Martiny L, Paulson OB, Knudsen GM. 2004. A database of [(18)F]-altanserin binding to 5-HT(2A) receptors in normal volunteers: normative data and relationship to physiological and demographic variables. Neuroimage 21:1105–1113.

Amodio DM, Frith CD. 2006. Meeting of minds: the medial frontal cortex and social cognition. Nat Rev Neurosci 7:268–277.

Bailer UF, Frank GK, Henry SE, Price JC, Meltzer CC, Weissfeld L, Mathis CA, Drevets WC, Wagner A, Hoge J, Ziolko SK, McConaha CW, Kaye WH. 2005. Altered brain serotonin 5-HT1A receptor binding after recovery from anorexia nervosa measured by positron emission tomography and [carbonyl11C]WAY-100635. Arch Gen Psychiatry 62:1032–1041.

Bailer UF, Frank GK, Henry SE, Price JC, Meltzer CC, Mathis CA, Wagner A, Thornton L, Hoge J, Ziolko SK, Becker CR, McConaha CW, Kaye WH. 2007. Exaggerated 5-HT1A but normal 5-HT2A receptor activity in individuals ill with anorexia nervosa. Biol Psychiatry 61:1090–1099.

Bech P. 1988. Rating scales for mood disorders: applicability, consistency and construct validity. Acta Psychiatr Scand 78(Suppl 345): 45–55.

Bech P, Kastrup M, Rafaelsen OJ. 1986. Mini-compendium of rating scales for states of anxiety, depression, mania, schizophrenia with corresponding DSM-III syndromes. Acta Psychiatr Scand 73 (Suppl. 326):6–37.

Bhagwagar Z, Hinz R, Taylor M, Fancy S, Cowen P, Grasby P. 2006. Increased 5-HT2A receptor binding in euthymic. medication-free patients recovered from depression: A positron emission study with [11C]MDL 100,907. Am J Psychiatry 163:1580–1587.

Borg J, Andrée B, Soderstrom H, Farde L. 2003. The serotonin system and spiritual experiences. Am J Psychiatry 160:1965–1969.

Damasio AR. 1994. Descartes' error: Emotion, reason, and the human brain. New York: Putnam.

Duman RS. 2004. The neurochemistry of depressive disorders: Preclinical studies, in neurobiology of mental illness. In: Charney D, Nestler EJ, editors. New York: Oxford University Press. p 421–439

Folstein MF, Folstein SE, McHugh PR. 1975. 'Minimental state'—A practical method for grading the cognitive state of patients for the clinician. J Psychiatr Res 12:189–198.

Geday J, Hermansen F, Rosenberg R, Smith DF. 2005. Serotonin modulation of cerebral blood flow measured with positron emission tomography (PET) in humans. Synapse 55:224–229.

Geday J, Ostergaard K, Gjedde A. 2006. Stimulation of subthalamic nucleus inhibits emotional activation of fusiform gyrus. Neuroimage 33:706–714.

Geday J, Kupers R, Gjedde A. 2007. As time goes by: Temporal constraints on emotional activation of inferior medial prefrontal cortex. Cereb Cortex 17:2753–2759.

Graff-Guerrero A, De la Fuente-Sandoval C, Camarena B, Gomez-Martin D, Apiquia R, Fresa A, Aguilar A, Mendez-Nunez JC, Escalona-Huerta C, Drucker-Colı R, Nicolinie H. 2005. Frontal and limbic metabolic differences in subjects selected according to genetic variation of the SLC6A4 gene polymorphism. Neuroimage 25:1197–1204.

Greenberg BD, Li Q, Lucas FR, Hu S, Sirota LA, Benjamin J, Lesch KP, Hamer D, Murphy DL. 2000. Association between the serotonin transporter promoter polymorphism and personality traits in a primarily female population sample. Am J Med Genet 96:202–216.

Grosbras MH, Paus T. 2006. Brain networks involved in viewing angry hands or faces. Cereb Cortex 16:1087–1096.

Harvey PO, Fossati P, Lepage M. 2007a. Modulation of memory formation by stimulus content: Specific role of the medial prefrontal cortex in the successful encoding of social pictures. Cogn Neurosci 19:351–362.

Harvey PO, Pruessner J, Czechowska Y, Lepage M. 2007b. Individual differences in trait anhedonia: A structural and functional magnetic resonance imaging study in non-clinical subjects. Mol Psychiatry 12:703, 767–775.

Iidaka T, Omori M, Murata T, Kosaka H, Yonekura Y, Okada T, Sadato N. 2001. Neural interaction of the amygdala with the prefrontal and temporal cortices in the processing of facial expressions as revealed by fMRI. J Cogn Neurosci 13:1035–1047.

Lang PJ, Bradley MM, Cuthbert BN. 2005. International affective picture system (IAPS): Affective ratings of pictures and instruction manual. Technical Report A-6. University of Florida, Gainesville, FL.

Lesch KP, Mossner R. 1998. Genetically driven variation in serotonin uptake: Is there a link to affective spectrum, neurodevelopmental, and neurodegenerative disorders? Biol Psychiatry 44:179–192.

Lesch KP, Bengel D, Heils A, Sabol SZ, Greenberg BD, Petri S, Benjamin J, Muller CR, Hamer DH, Murphy DL. 1996. Association of anxiety-related traits with a polymorphism in the serotonin transporter gene regulatory region. Science 274:1527–1531.

Mäestu J, Allik J, Merenäkk L, Eensoo D, Parik J, Veidebaum T, Harro J. 2008. Associations between an α_{2a} adrenergic receptor gene polymorphism and adolescent personality. Am J Med Genet B Neuropsychiatr Genet. Am J Med Genet B Neuropsychiatr Genet 147B:418–423.

Moresco FM, Dieci M, Vita A, Messa C, Gobbo C, Galli L, Rizzo G, Panzacchi A, De Peri L, Invernizzi G, Fazio F. 2002. In Vivo serotonin 5HT2A receptor binding and personality traits in healthy subjects: A positron emission tomography study. NeuroImage 17:1470–1478.

Møller M, Jakobsen S, Gjedde A. 2007. Parametric and regional maps of free serotonin 5HT1A receptor sites in human brain as function of age in healthy humans. Neuropsychopharmacology. 32:1707–1714.
Neumeister A, Drevets WC, Belfer I, Luckenbaugh DA, Henry S, Bonne O, Herscovitch P, Goldman D, Charney DS. 2006. Effects of a alpha 2C-adrenoreceptor gene polymorphism on neural responses to facial expressions in depression. Neuropsychopharmacology 31:1750–1756.
Ochsner KN, Gross JJ. 2005. The cognitive control of emotion. Trends Cogn Sci 9:242–249.
Oquendo MA, Krunic A, Parsey RV, Milak M, Malone KM, Anderson A, van Heertum RL, John Mann J. 2005. Positron emission tomography of regional brain metabolic responses to a serotonergic challenge in major depressive disorder with and without borderline personality disorder. Neuropsychopharmacology 30:1163–1172.
Paradiso S, Johnson DL, Andreasen NC, O'Leary DS, Watkins GL, Ponto LL, Hichwa RD. 1999. Cerebral blood flow changes associated with attribution of emotional valence to pleasant, unpleasant, and neutral visual stimuli in a PET study of normal subjects. Am J Psychiatry 156:1618–1629.
Peroutka SJ, Snyder SH. 1983. Multiple serotonin receptors and their physiological significance. Fed Proc 42:213–217.
Phan KL, Wager T, Taylor SF, Liberzon J. 2002. Functional neuroanatomy of emotion: A meta-analysis of emotion activation studies in PET and fMRI. Neuroimage 16:331–348.
Phillips ML, Bullmore ET, Howard R, Woodruff PW, Wright IC, Williams SC, Simmons A, Andrew C, Brammer M, David AS. 1998. Investigation of facial recognition memory and happy and sad facial expression perception: an fMRI study. Psychiatry Res 83:127–138.
Raichle ME, MacLeod AM, Snyder AZ, Powers WJ, Gusnard DA, Shulman GL. 2001. A default mode of brain function. Proc Natl Acad Sci USA 98:676–682.
Ramos BP, Arnsten AF. 2007. Adrenergic pharmacology and cognition: focus on the prefrontal cortex. Pharmacol Ther 113:523–536.
Schilbach L, Wohlschlaeger AM, Kraemer NC, Newen A, Shah NJ, Fink GR, Vogeley K. 2006. Being with virtual others: Neural correlates of social interaction. Neuropsychologia 44:718–730.
Smith DF, Geday J. 2001. PET neuroimaging of clomipramine challenge in humans: Focus on the thalamus. Brain Res 892:193–197.
Soloff PH, Meltzer CC, Greer PJ, Constantine D, Kelly TM. 2000. A fenfluramine-activated FDG-PET study of borderline personality disorder. Biol Psychiatry 47:540–547.
Talairach J, Tournoux P. 1988. A Co-planar stereotaxic atlas of the human brain. Stuttgart: Thieme.
Worsley KJ, Marrett S, Neelin P, Vandal AC, Friston KJ, Evans AC. 1996. A unified statistical approach for determining significant signals in images of cerebral activation. Hum Brain Mapp 4:458–473.

APPENDIX

The reactivity of a receptor system is the incremental binding of neurotransmitters obtained with a unit increase of transmitter concentration, in the simplest case equal to the slope of the Michaelis-Menten curve at any concentration. The reactivity is the magnitude of this slope,

$$R = \frac{B_{\max} K_{\mathrm{d}}}{(K_{\mathrm{d}} + C)^2} \quad \text{(A1)}$$

where, R is the reactivity, $B_{\max}$ the receptor density, K_{d} is the affinity towards the transmitter, and C is the transmitter's concentration. Thus, the incremental binding leads to increased excitation if the receptor subtype mediates excitation, and it leads to increased inhibition if the receptor subtype mediates inhibition. If two kinetically different receptor subtypes of opposite action both respond to the increase of transmitter, then the net effect depends on the concentration of the transmitter. At a specific concentration (C_0) the effect of inhibitory and excitatory actions are then equally large,

$$C_o = K_{\mathrm{e}} \frac{\sqrt{R_{\mathrm{B}} R_{\mathrm{K}}} - R_{\mathrm{K}}}{1 - \sqrt{R_{\mathrm{B}} R_{\mathrm{K}}}} \quad \text{(A2)}$$

where, C_0 is the threshold concentration of the transmitter, K_{e} is the affinity of the receptor mediating excitation, R_{B} is the ratio of inhibitory to excitatory receptor densities, and R_{K} is the ratio of inhibitory to excitatory receptor affinities. Below the threshold concentration, the net effect of a transmitter surge is inhibitory, while above this concentration the net effect is excitatory, as shown in Figure A1. In turn, if different individuals react differently to the same stimuli, at different times or because of individual differences of receptor properties, one explanation is different baseline monoamine levels, respectively below and above the C_0 value derived above.

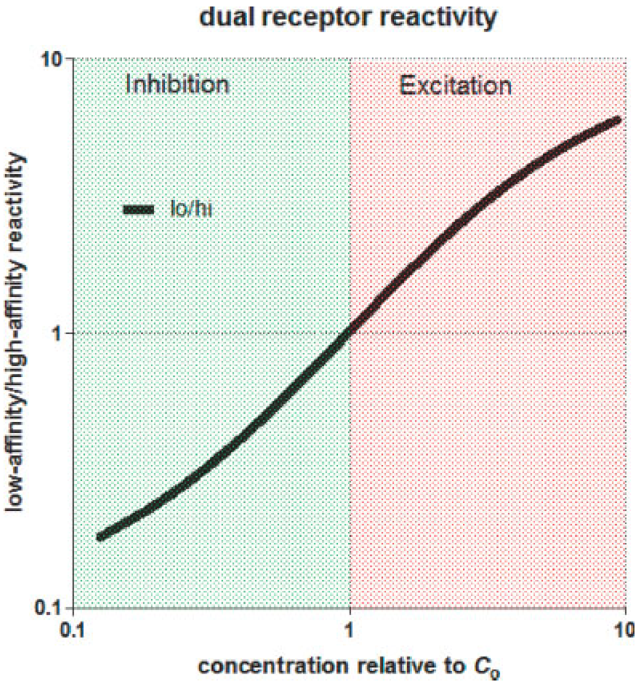

Fig. A1. Relationship between net excitation or inhibition as function of transmitter concentration for regions of monoaminergic neurotransmission targeting at least receptor subtypes of different density, affinity, and action, as predicted by Eqs. A1 and A2. Abscissa: Concentration of transmitter (e.g., serotonin and/or noradrenaline) relative to threshold concentration according to Eq. A2. Ordinate: Net effect (excitation or inhibition) reflected in proportional change of blood flow elicited by the respective coincentrations of monoamine. Axes are logarithmic. Graph predicts, as example, for baseline serotonin concentrations below C_0, given the properties of the serotonin receptor subtypes in IMPC, that an SNRI /SSRI induced increment of serotonin concentration yields a decrease of rCBF, while for baseline serotonin concentrations above C_0, the challenge leads to an increase of rCBF. The threshold concentration depends on the local $5HT_{1A}/5HT_{2A}$ receptor ratios, as indicated in Eq. A2.
